# 3-Ethyl­sulfanyl-2-(4-fluoro­phen­yl)-5-phenyl-1-benzofuran

**DOI:** 10.1107/S1600536810000760

**Published:** 2010-01-13

**Authors:** Hong Dae Choi, Pil Ja Seo, Byeng Wha Son, Uk Lee

**Affiliations:** a Department of Chemistry, Dongeui University, San 24 Kaya-dong Busanjin-gu, Busan 614-714, Republic of Korea; b Department of Chemistry, Pukyong National University, 599-1 Daeyeon 3-dong, Nam-gu, Busan 608-737, Republic of Korea

## Abstract

In the title compound, C_22_H_17_FOS, the crystal studied was an inversion twin with a 0.42 (18):0.58 (18) domain ratio. The 4-fluoro­phenyl ring is rotated out of the benzofuran plane, making a dihedral angle of 17.82 (6)°, and the dihedral angle between the 5-phenyl ring and the benzofuran plane is 29.45 (7)°.

## Related literature

For the crystal structures of similar 2,5-diaryl-1-benzofuran derivatives, see: Choi *et al.* (2006 ▶, 2009 ▶). For natural products with benzofuran rings, see: Akgul & Anil (2003 ▶); Soekamto *et al.* (2003 ▶); von Reuss & König (2004 ▶).
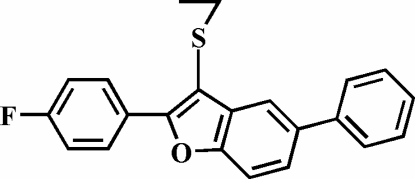


## Experimental

### 

#### Crystal data

C_22_H_17_FOS
*M* *_r_* = 348.42
Monoclinic, 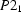

*a* = 10.5799 (2) Å
*b* = 7.1788 (1) Å
*c* = 11.9361 (2) Å
β = 110.031 (1)°
*V* = 851.72 (2) Å^3^
*Z* = 2
Mo *K*α radiation
μ = 0.21 mm^−1^
*T* = 173 K
0.26 × 0.23 × 0.20 mm

#### Data collection

Bruker SMART APEXII CCD diffractometer
Absorption correction: multi-scan (*SADABS*; Bruker, 2009 ▶) *T*_min_ = 0.685, *T*_max_ = 0.746
15026 measured reflections
2121 independent reflections
2068 reflections with *I* > 2σ(*I*)
*R*_int_ = 0.027

#### Refinement

*R*[*F* ^2^ > 2σ(*F* ^2^)] = 0.030
*wR*(*F* ^2^) = 0.079
*S* = 1.14
2121 reflections
227 parameters
1 restraint
H-atom parameters constrained
Δρ_max_ = 0.22 e Å^−3^
Δρ_min_ = −0.24 e Å^−3^
Absolute structure: Flack (1983 ▶), 1709 Friedel pairs
Flack parameter: 0.42 (18)

### 

Data collection: *APEX2* (Bruker, 2009 ▶); cell refinement: *SAINT* (Bruker, 2009 ▶); data reduction: *SAINT*; program(s) used to solve structure: *SHELXS97* (Sheldrick, 2008 ▶); program(s) used to refine structure: *SHELXL97* (Sheldrick, 2008 ▶); molecular graphics: *ORTEP-3* (Farrugia, 1997 ▶) and *DIAMOND* (Brandenburg, 1998 ▶); software used to prepare material for publication: *SHELXL97*.

## Supplementary Material

Crystal structure: contains datablocks global, I. DOI: 10.1107/S1600536810000760/ng2718sup1.cif

Structure factors: contains datablocks I. DOI: 10.1107/S1600536810000760/ng2718Isup2.hkl

Additional supplementary materials:  crystallographic information; 3D view; checkCIF report

## Figures and Tables

**Fig. 1.**
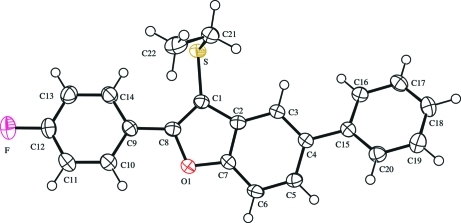
The molecular structure of the title compound with the atom numbering scheme. Displacement ellipsoids are drawn at the 50 % probability level. H atoms are presented as a small cycles of arbitrary radius.

## supplementary crystallographic information

### Comment

Benzofuran compounds are considerable interesting heterocycles, which are occurring in nature and show diverse biological activities (Akgul & Anil, 2003; Soekamto *et al.*, 2003; von Reuss & König, 2004). As a part of our continuing studies of the effect of side chain substituents on the solid state structures of 2,5-diaryl-1-benzofuran analogues (Choi *et al.*, 2006, 2009), we report the crystal structure of the title compound (Fig. 1).

The title compound crystallizes as the monoclinic space P21. The crystal studied was an inversion twin with a 0.42 (18) : 0.58 (18) domain ratio. The benzofuran unit is essentially planar, with a mean deviation of 0.019 (1) Å from the least-squares plane defined by the nine constituent atoms. The 4-fluorophenyl ring is rotated out of the benzofuran plane, with a dihedral angle of 17.82 (6)°. The dihedral angle between the phenyl ring and the benzofuran plane is 29.45 (7)°.

### Experimental

Zinc chloride (273 mg, 2.0 mmol) was added to a stirred solution of 4-phenylphenol (340 mg, 2.0 mmol) and 2-chloro-2-ethylsulfanyl-4'-fluoroacetophenone (465 mg, 2.0 mmol) in dichloromethane (25 ml) at room temperature, and stirring was continued at the same temperature for 40 min. The reaction was quenched by the addition of water and the organic layer separated, dried over magnesium sulfate, filtered and concentrated in vacuum. The residue was purified by column chromatography (carbon tetrachloride) to afford the title compound as a colorless solid [yield 66 %, m.p. 393-394 K; *R*_f_ = 0.76 (carbon tetrachloride)]. Single crystals suitable for X-ray diffraction were prepared by evaporation of a solution of the title compound in acetone at room temperature.

### Refinement

The reported Flack parameter was obtained by TWIN/BASF procedure in SHELXL (Sheldrick, 2008). All H atoms were geometrically positioned and refined using a riding model, with C–H = 0.95 Å for aryl, 0.99 Å for methylene, and 0.98 Å for methyl H atoms. *U*_iso_(H) = 1.2*U*_eq_(C) for all H atoms.

### Crystal data

**Table:**

C_22_H_17_FOS	*F*(000) = 364
*M**_r_* = 348.42	*D*_x_ = 1.359 Mg m^−^^3^
Monoclinic, *P*2_1_	Mo *K*α radiation, λ = 0.71073 Å
Hall symbol: P 2yb	Cell parameters from 9177 reflections
*a* = 10.5799 (2) Å	θ = 2.2–27.5°
*b* = 7.1788 (1) Å	µ = 0.21 mm^−^^1^
*c* = 11.9361 (2) Å	*T* = 173 K
β = 110.031 (1)°	Block, colourless
*V* = 851.72 (2) Å^3^	0.26 × 0.23 × 0.20 mm
*Z* = 2	

### Data collection

**Table:**

Bruker SMART APEXII CCD diffractometer	2121 independent reflections
Radiation source: Rotating Anode	2068 reflections with *I* > 2σ(*I*)
HELIOS	*R*_int_ = 0.027
Detector resolution: 10.0 pixels mm^-1^	θ_max_ = 27.6°, θ_min_ = 1.8°
φ and ω scans	*h* = −13→13
Absorption correction: multi-scan (*SADABS*; Bruker, 2009)	*k* = −9→8
*T*_min_ = 0.685, *T*_max_ = 0.746	*l* = −14→15
15026 measured reflections	

### Refinement

**Table:**

Refinement on *F*^2^	Secondary atom site location: difference Fourier map
Least-squares matrix: full	Hydrogen site location: difference Fourier map
*R*[*F*^2^ > 2σ(*F*^2^)] = 0.030	H-atom parameters constrained
*wR*(*F*^2^) = 0.079	*w* = 1/[σ^2^(*F*_o_^2^) + (0.0453*P*)^2^ + 0.1397*P*] where *P* = (*F*_o_^2^ + 2*F*_c_^2^)/3
*S* = 1.14	(Δ/σ)_max_ < 0.001
2121 reflections	Δρ_max_ = 0.22 e Å^−^^3^
227 parameters	Δρ_min_ = −0.24 e Å^−^^3^
1 restraint	Absolute structure: Flack (1983), 1709 Friedel pairs
Primary atom site location: structure-invariant direct methods	Flack parameter: 0.42 (18)

### Special details

**Table:**

Geometry. All esds (except the esd in the dihedral angle between two l.s. planes) are estimated using the full covariance matrix. The cell esds are taken into account individually in the estimation of esds in distances, angles and torsion angles; correlations between esds in cell parameters are only used when they are defined by crystal symmetry. An approximate (isotropic) treatment of cell esds is used for estimating esds involving l.s. planes.
Refinement. Refinement of F^2^ against ALL reflections. The weighted R-factor wR and goodness of fit S are based on F^2^, conventional R-factors R are based on F, with F set to zero for negative F^2^. The threshold expression of F^2^ > 2sigma(F^2^) is used only for calculating R-factors(gt) etc. and is not relevant to the choice of reflections for refinement. R-factors based on F^2^ are statistically about twice as large as those based on F, and R- factors based on ALL data will be even larger.

### Fractional atomic coordinates and isotropic or equivalent isotropic displacement parameters (Å^2^)

**Table:**

	*x*	*y*	*z*	*U*_iso_*/*U*_eq_	
S	0.50045 (5)	0.02884 (16)	0.84349 (4)	0.03160 (13)	
O1	0.63181 (12)	0.1017 (2)	0.57201 (11)	0.0289 (3)	
F	1.19038 (12)	0.1458 (3)	0.98123 (13)	0.0484 (4)	
C1	0.53216 (18)	0.0628 (3)	0.71024 (16)	0.0262 (4)	
C2	0.42846 (18)	0.0729 (3)	0.59439 (15)	0.0258 (4)	
C3	0.28812 (17)	0.0710 (3)	0.55375 (16)	0.0259 (4)	
H3	0.2411	0.0561	0.6082	0.031*	
C4	0.21795 (18)	0.0913 (3)	0.43231 (16)	0.0251 (4)	
C5	0.29034 (19)	0.1086 (3)	0.35301 (17)	0.0289 (4)	
H5	0.2419	0.1183	0.2699	0.035*	
C6	0.42947 (19)	0.1118 (3)	0.39222 (17)	0.0301 (4)	
H6	0.4773	0.1244	0.3382	0.036*	
C7	0.49542 (19)	0.0960 (3)	0.51340 (17)	0.0270 (4)	
C8	0.65154 (19)	0.0828 (3)	0.69190 (16)	0.0269 (4)	
C9	0.79275 (18)	0.0965 (3)	0.76965 (17)	0.0277 (4)	
C10	0.88722 (19)	0.1718 (3)	0.72355 (19)	0.0306 (4)	
H10	0.8591	0.2111	0.6427	0.037*	
C11	1.0211 (2)	0.1894 (4)	0.7948 (2)	0.0350 (5)	
H11	1.0851	0.2415	0.7641	0.042*	
C12	1.05873 (19)	0.1296 (3)	0.91040 (19)	0.0343 (5)	
C13	0.9699 (2)	0.0546 (4)	0.95918 (18)	0.0373 (5)	
H13	0.9997	0.0148	1.0400	0.045*	
C14	0.83584 (19)	0.0383 (4)	0.88783 (18)	0.0348 (4)	
H14	0.7729	−0.0130	0.9200	0.042*	
C15	0.06821 (17)	0.1006 (3)	0.38741 (16)	0.0249 (4)	
C16	−0.00642 (18)	0.0113 (3)	0.44819 (17)	0.0286 (4)	
H16	0.0385	−0.0622	0.5164	0.034*	
C17	−0.14547 (18)	0.0287 (4)	0.40998 (18)	0.0331 (4)	
H17	−0.1949	−0.0315	0.4527	0.040*	
C18	−0.2123 (2)	0.1335 (4)	0.3099 (2)	0.0363 (5)	
H18	−0.3073	0.1466	0.2844	0.044*	
C19	−0.1402 (2)	0.2190 (4)	0.2472 (2)	0.0368 (5)	
H19	−0.1860	0.2889	0.1775	0.044*	
C20	−0.0015 (2)	0.2032 (3)	0.28564 (18)	0.0307 (4)	
H20	0.0471	0.2632	0.2420	0.037*	
C21	0.4401 (2)	0.2604 (4)	0.86144 (19)	0.0361 (5)	
H21A	0.3691	0.2976	0.7862	0.043*	
H21B	0.3995	0.2568	0.9248	0.043*	
C22	0.5514 (3)	0.4048 (4)	0.8936 (2)	0.0433 (6)	
H22A	0.5902	0.4116	0.8300	0.052*	
H22B	0.6215	0.3694	0.9686	0.052*	
H22C	0.5145	0.5266	0.9030	0.052*	

### Atomic displacement parameters (Å^2^)

**Table:**

	*U*^11^	*U*^22^	*U*^33^	*U*^12^	*U*^13^	*U*^23^
S	0.0324 (2)	0.0376 (3)	0.0280 (2)	−0.0010 (2)	0.01449 (17)	0.0058 (2)
O1	0.0248 (6)	0.0356 (8)	0.0288 (6)	0.0005 (6)	0.0122 (5)	0.0003 (6)
F	0.0268 (6)	0.0558 (10)	0.0544 (8)	−0.0006 (6)	0.0034 (5)	0.0004 (7)
C1	0.0282 (8)	0.0254 (11)	0.0271 (8)	0.0006 (7)	0.0121 (7)	0.0024 (8)
C2	0.0308 (9)	0.0228 (10)	0.0263 (8)	−0.0002 (7)	0.0129 (7)	−0.0009 (7)
C3	0.0273 (8)	0.0256 (11)	0.0277 (8)	−0.0010 (7)	0.0133 (7)	−0.0004 (8)
C4	0.0265 (8)	0.0217 (9)	0.0288 (8)	−0.0015 (7)	0.0117 (7)	−0.0014 (8)
C5	0.0328 (9)	0.0305 (10)	0.0249 (8)	−0.0011 (8)	0.0117 (7)	−0.0019 (8)
C6	0.0324 (9)	0.0344 (11)	0.0290 (9)	−0.0016 (9)	0.0177 (7)	−0.0008 (9)
C7	0.0253 (7)	0.0273 (9)	0.0311 (9)	−0.0004 (8)	0.0133 (7)	−0.0011 (8)
C8	0.0303 (8)	0.0245 (10)	0.0283 (8)	0.0019 (7)	0.0131 (7)	0.0002 (8)
C9	0.0256 (8)	0.0244 (9)	0.0344 (9)	0.0021 (8)	0.0120 (7)	−0.0005 (8)
C10	0.0294 (9)	0.0272 (10)	0.0361 (10)	0.0021 (8)	0.0124 (7)	0.0030 (9)
C11	0.0283 (9)	0.0319 (11)	0.0483 (12)	0.0001 (8)	0.0175 (9)	0.0021 (10)
C12	0.0250 (9)	0.0302 (12)	0.0433 (11)	0.0018 (8)	0.0062 (8)	−0.0024 (9)
C13	0.0349 (10)	0.0405 (15)	0.0330 (10)	0.0042 (10)	0.0072 (8)	0.0028 (10)
C14	0.0305 (9)	0.0375 (12)	0.0374 (9)	−0.0001 (10)	0.0131 (7)	0.0043 (10)
C15	0.0261 (8)	0.0220 (9)	0.0277 (8)	−0.0020 (7)	0.0105 (7)	−0.0044 (8)
C16	0.0309 (8)	0.0259 (10)	0.0310 (8)	−0.0015 (9)	0.0131 (7)	−0.0001 (9)
C17	0.0314 (9)	0.0309 (10)	0.0416 (10)	−0.0051 (10)	0.0184 (7)	−0.0042 (11)
C18	0.0245 (8)	0.0356 (13)	0.0460 (11)	−0.0019 (8)	0.0087 (8)	−0.0036 (10)
C19	0.0338 (10)	0.0332 (12)	0.0378 (11)	−0.0010 (9)	0.0049 (8)	0.0034 (9)
C20	0.0330 (10)	0.0285 (11)	0.0317 (10)	−0.0037 (8)	0.0123 (8)	0.0014 (8)
C21	0.0332 (10)	0.0446 (13)	0.0331 (10)	0.0050 (9)	0.0148 (8)	−0.0020 (10)
C22	0.0565 (14)	0.0429 (14)	0.0339 (11)	−0.0064 (11)	0.0197 (10)	−0.0062 (10)

### Geometric parameters (Å, °)

**Table:**

S—C1	1.751 (2)	C11—H11	0.9500
S—C21	1.819 (3)	C12—C13	1.373 (3)
O1—C7	1.372 (2)	C13—C14	1.387 (3)
O1—C8	1.380 (2)	C13—H13	0.9500
F—C12	1.364 (2)	C14—H14	0.9500
C1—C8	1.361 (3)	C15—C16	1.398 (3)
C1—C2	1.443 (3)	C15—C20	1.396 (3)
C2—C7	1.390 (2)	C16—C17	1.389 (2)
C2—C3	1.395 (2)	C16—H16	0.9500
C3—C4	1.391 (2)	C17—C18	1.383 (3)
C3—H3	0.9500	C17—H17	0.9500
C4—C5	1.412 (2)	C18—C19	1.381 (3)
C4—C15	1.490 (2)	C18—H18	0.9500
C5—C6	1.384 (3)	C19—C20	1.384 (3)
C5—H5	0.9500	C19—H19	0.9500
C6—C7	1.379 (3)	C20—H20	0.9500
C6—H6	0.9500	C21—C22	1.516 (3)
C8—C9	1.467 (3)	C21—H21A	0.9900
C9—C14	1.390 (3)	C21—H21B	0.9900
C9—C10	1.404 (3)	C22—H22A	0.9800
C10—C11	1.386 (3)	C22—H22B	0.9800
C10—H10	0.9500	C22—H22C	0.9800
C11—C12	1.368 (3)		
			
C1—S—C21	99.43 (12)	C11—C12—C13	123.07 (18)
C7—O1—C8	106.56 (14)	C12—C13—C14	118.5 (2)
C8—C1—C2	106.46 (16)	C12—C13—H13	120.8
C8—C1—S	129.62 (15)	C14—C13—H13	120.8
C2—C1—S	123.92 (13)	C13—C14—C9	120.58 (19)
C7—C2—C3	119.63 (16)	C13—C14—H14	119.7
C7—C2—C1	105.73 (16)	C9—C14—H14	119.7
C3—C2—C1	134.59 (16)	C16—C15—C20	118.04 (16)
C4—C3—C2	119.06 (16)	C16—C15—C4	120.86 (17)
C4—C3—H3	120.5	C20—C15—C4	121.07 (17)
C2—C3—H3	120.5	C17—C16—C15	120.76 (19)
C3—C4—C5	119.28 (16)	C17—C16—H16	119.6
C3—C4—C15	120.10 (16)	C15—C16—H16	119.6
C5—C4—C15	120.59 (16)	C18—C17—C16	120.19 (19)
C6—C5—C4	122.21 (17)	C18—C17—H17	119.9
C6—C5—H5	118.9	C16—C17—H17	119.9
C4—C5—H5	118.9	C17—C18—C19	119.72 (18)
C7—C6—C5	116.81 (16)	C17—C18—H18	120.1
C7—C6—H6	121.6	C19—C18—H18	120.1
C5—C6—H6	121.6	C20—C19—C18	120.2 (2)
O1—C7—C6	126.77 (16)	C20—C19—H19	119.9
O1—C7—C2	110.27 (16)	C18—C19—H19	119.9
C6—C7—C2	122.96 (18)	C19—C20—C15	121.02 (18)
C1—C8—O1	110.98 (16)	C19—C20—H20	119.5
C1—C8—C9	134.83 (18)	C15—C20—H20	119.5
O1—C8—C9	114.13 (15)	C22—C21—S	112.39 (16)
C14—C9—C10	118.91 (17)	C22—C21—H21A	109.1
C14—C9—C8	122.06 (17)	S—C21—H21A	109.1
C10—C9—C8	119.03 (18)	C22—C21—H21B	109.1
C11—C10—C9	120.7 (2)	S—C21—H21B	109.1
C11—C10—H10	119.7	H21A—C21—H21B	107.9
C9—C10—H10	119.7	C21—C22—H22A	109.5
C12—C11—C10	118.28 (19)	C21—C22—H22B	109.5
C12—C11—H11	120.9	H22A—C22—H22B	109.5
C10—C11—H11	120.9	C21—C22—H22C	109.5
F—C12—C11	118.76 (19)	H22A—C22—H22C	109.5
F—C12—C13	118.17 (19)	H22B—C22—H22C	109.5
			
C21—S—C1—C8	105.3 (2)	C1—C8—C9—C14	18.7 (4)
C21—S—C1—C2	−75.37 (19)	O1—C8—C9—C14	−164.5 (2)
C8—C1—C2—C7	0.9 (2)	C1—C8—C9—C10	−160.9 (2)
S—C1—C2—C7	−178.61 (17)	O1—C8—C9—C10	15.9 (3)
C8—C1—C2—C3	−176.4 (2)	C14—C9—C10—C11	−0.5 (3)
S—C1—C2—C3	4.1 (3)	C8—C9—C10—C11	179.1 (2)
C7—C2—C3—C4	0.4 (3)	C9—C10—C11—C12	0.7 (3)
C1—C2—C3—C4	177.4 (2)	C10—C11—C12—F	179.8 (2)
C2—C3—C4—C5	1.6 (3)	C10—C11—C12—C13	−0.5 (4)
C2—C3—C4—C15	−176.53 (18)	F—C12—C13—C14	179.8 (2)
C3—C4—C5—C6	−2.0 (3)	C11—C12—C13—C14	0.1 (4)
C15—C4—C5—C6	176.1 (2)	C12—C13—C14—C9	0.1 (4)
C4—C5—C6—C7	0.4 (3)	C10—C9—C14—C13	0.1 (4)
C8—O1—C7—C6	179.2 (2)	C8—C9—C14—C13	−179.5 (2)
C8—O1—C7—C2	−0.3 (2)	C3—C4—C15—C16	−28.7 (3)
C5—C6—C7—O1	−177.8 (2)	C5—C4—C15—C16	153.2 (2)
C5—C6—C7—C2	1.6 (3)	C3—C4—C15—C20	149.3 (2)
C3—C2—C7—O1	177.43 (17)	C5—C4—C15—C20	−28.8 (3)
C1—C2—C7—O1	−0.4 (2)	C20—C15—C16—C17	−1.7 (3)
C3—C2—C7—C6	−2.1 (3)	C4—C15—C16—C17	176.4 (2)
C1—C2—C7—C6	−179.9 (2)	C15—C16—C17—C18	0.8 (4)
C2—C1—C8—O1	−1.1 (2)	C16—C17—C18—C19	0.8 (4)
S—C1—C8—O1	178.35 (17)	C17—C18—C19—C20	−1.3 (4)
C2—C1—C8—C9	175.8 (2)	C18—C19—C20—C15	0.3 (3)
S—C1—C8—C9	−4.8 (4)	C16—C15—C20—C19	1.2 (3)
C7—O1—C8—C1	0.9 (2)	C4—C15—C20—C19	−176.9 (2)
C7—O1—C8—C9	−176.69 (18)	C1—S—C21—C22	−70.70 (17)

## References

[bb1] Akgul, Y. Y. & Anil, H. (2003). *Phytochemistry*, **63**, 939–943. 10.1016/s0031-9422(03)00357-1 12895543

[bb2] Brandenburg, K. (1998). *DIAMOND* Crystal Impact GbR, Bonn, Germany.

[bb3] Bruker (2009). *APEX2* and *SAINT* Bruker AXS Inc., Madison, Wisconsin, USA.

[bb4] Choi, H. D., Seo, P. J., Kang, B. W., Son, B. W. & Lee, U. (2006). *Acta Cryst.* E**62**, o4796–o4797.

[bb5] Choi, H. D., Seo, P. J., Son, B. W. & Lee, U. (2009). *Acta Cryst.* E**65**, o2766. 10.1107/S1600536809041713 PMC2971277 21578360

[bb6] Farrugia, L. J. (1997). *J. Appl. Cryst.* **30**, 565.

[bb7] Flack, H. D. (1983). *Acta Cryst.* A**39**, 876–881.

[bb8] Reuss, S. H. von & König, W. A. (2004). *Phytochemistry*, **65**, 3113–3118. 10.1016/j.phytochem.2004.10.002 15541739

[bb9] Sheldrick, G. M. (2008). *Acta Cryst.* A**64**, 112–122. 10.1107/S0108767307043930 18156677

[bb10] Soekamto, N. H., Achmad, S. A., Ghisalberti, E. L., Hakim, E. H. & Syah, Y. M. (2003). *Phytochemistry*, **64**, 831–834. 10.1016/j.phytochem.2003.08.009 14559276

